# Harm minimisation for the management of self-harm: a mixed-methods analysis of electronic health records in secondary mental healthcare

**DOI:** 10.1192/bjo.2021.946

**Published:** 2021-06-25

**Authors:** Charlotte Cliffe, Alexandra Pitman, Rosemary Sedgwick, Megan Pritchard, Rina Dutta, Sarah Rowe

**Affiliations:** NIHR Biomedical Research Centre, King’s College London and SLaM NHS Trust, UK; and UCL Division of Psychiatry, UCL, UK; UCL Division of Psychiatry, UCL, UK; and Camden & Islington NHS Foundation Trust, UK; NIHR Biomedical Research Centre, King’s College London and SLaM NHS Trust, UK; NIHR Biomedical Research Centre, King’s College London and SLaM NHS Trust, UK; NIHR Biomedical Research Centre, King’s College London and SLaM NHS Trust, UK; UCL Division of Psychiatry, UCL, UK

**Keywords:** Self-harm, non-suicidal self-injury, harm minimisation, harm reduction

## Abstract

**Background:**

Prevalence of self-harm in the UK was reported as 6.4% in 2014. Despite sparse evidence for effectiveness, guidelines recommend harm minimisation; a strategy in which people who self-harm are supported to do so safely.

**Aims:**

To determine the prevalence, sociodemographic and clinical characteristics of those who self-harm and practise harm minimisation within a London mental health trust.

**Method:**

We included electronic health records for patients treated by South London and Maudsley NHS Trust. Using an iterative search strategy, we identified patients who practise harm minimisation, then classified the approaches using a content analysis. We compared the sociodemographic characteristics with that of a control group of patients who self-harm and do not use harm minimisation.

**Results:**

In total 22 736 patients reported self-harm, of these 693 (3%) had records reporting the use of harm-minimisation techniques. We coded the approaches into categories: (a) ‘substitution’ (>50% of those using harm minimisation), such as using rubber bands or using ice; (b) ‘simulation’ (9%) such as using red pens; (c) ‘defer or avoid’ (7%) such as an alternative self-injury location; (d) ‘damage limitation’ (9%) such as using antiseptic techniques; the remainder were unclassifiable (24%). The majority of people using harm minimisation described it as helpful (>90%). Those practising harm minimisation were younger, female, of White ethnicity, had previous admissions and were less likely to have self-harmed with suicidal intent.

**Conclusions:**

A small minority of patients who self-harm report using harm minimisation, primarily substitution techniques, and the large majority find harm minimisation helpful. More research is required to determine the acceptability and effectiveness of harm-minimisation techniques and update national clinical guidelines.

## Background

Self-harm, defined as an intentional act of self-injury with or without suicidal intent, is a significant public health concern.^1^ Not only is self-harm associated with distress for individuals and their carers, but the estimated costs to the National Health Service (NHS) are over £160 million a year.^2^ The patient-reported prevalence of lifetime self-harm in community settings in 2014 was estimated at 6.4% in a representative English population sample, which has increased from 2.4% in the year 2000.^3^

Self-harm may cause permanent damage or physical complications such as infections, scarring or tendon damage and increases the risk of suicide.^4,5^ Many of those who self-harm use it is as a coping strategy to manage emotional dysregulation or underlying distress.^5,6^ There are evidence-based therapies that provide strategies to regulate these intense emotions and repetitive self-harm, such as cognitive–behavioural therapy (CBT) and dialectical behavioural therapy (DBT).^6–10^ However, there are long waiting times for DBT treatments, up to 2 years in the UK,^11,12^ and the drop-out rates are moderate, ranging from 26.2% for CBT and 28% for DBT.^13^ Although there is no trial evidence to support the practice of harm minimisation for self-harm, this is a strategy described in the National Institute for Health and Care Excellence (NICE) guidelines for the shorter-term management of self-harm (termed harm reduction).^14^ The guideline recommends reinforcing existing strategies and developing new strategies as an alternative to self-harm and to consider less destructive or harmful methods of self-harm.^14^

## Harm-minimisation strategies

Harm minimisation aims to reduce the negative impact of a behaviour without removing the behaviour completely^15,16^ if completely stopping the behaviour would potentially increase distress or project unrealistic expectations onto the individual.^14^ Harm-minimisation strategies are commonly practised in addiction services; this may include prescribing people who are opioid dependent methadone maintenance or encouraging clean needles.^15^ Harm-minimisation approaches within addiction services have been shown to improve outcomes such as treatment adherence^16,17^ and cost-effectiveness.^18^

Harm-minimisation practices for self-harm management range from pinging rubber bands and wound care to providing clean blades and education about safe anatomical positioning of self-cutting.^19,20^ Some of these harm-minimisation strategies for self-harm, such as clean blades have raised ethical concerns.^21^ Questions have been raised about an individual's capacity to make autonomous decisions about their self-harm while vulnerable and distressed.^22^ Some clinicians argue harm minimisation for self-harm runs counter to a professional's duty to protect the patient and ‘do no harm’.^22^ However, others argue that preventing individuals from any self-harm may result in increasing and overwhelming distress, escalating their behaviour and hampering recovery long term.^22,23^

## Research into harm minimisation

There have been no randomised controlled trials or large cohort studies investigating the outcomes of using harm minimisation for self-harm. The limited studies to date include an analysis of adolescent in-patient experiences, where harm-minimisation policies were perceived as positive in reducing self-harm, although some staff felt uncomfortable.^24^ An audit of psychiatric in-patients, where harm minimisation was practised, demonstrated that the frequency of self-harm recorded on admission had decreased on discharge.^25^ Although this study was not able to ascertain whether the change had been a direct result of the harm-minimisation policy, it demonstrated that in settings where harm minimisation was practised there was no increase in self-harm.^25^

A recent qualitative study with adolescents reported mixed views about whether harm minimisation was effective, but most adolescents described harm minimisation as ineffective.^26^ Another qualitative study analysed data with 11 adolescents and identified mixed views on harm minimisation; although some found that harm minimisation gave them greater competence at managing their self-harm, others described this as short lived, and expressed concerns about the potential for some individuals to misuse this knowledge.^27^ Conflicting findings from these few qualitative studies may reflect their low numbers of participants and specific sampling approaches; there is a need for larger-scale studies describing the prevalence and nature of the practice of harm minimisation in clinical services.^14,20^

## Aims

Our study aimed to determine the proportion of all patients within a large London secondary mental healthcare service who are documented as having used harm-minimisation techniques for self-harm, to describe the nature of harm-minimisation approaches used and compare the sociodemographic and clinical characteristics of patients who do and do not use harm-minimisation techniques for self-harm.

## Method

### Study design

#### Pilot study

A pilot study was undertaken using routine electronic health records of all patients based in a London mental health trust. Camden and Islington NHS Trust is a secondary mental health service covering 471 000 patients within the boroughs of Camden and Islington. The electronic health records for all patients from 2008 were available using a data extraction system, known as Clinical Records Interactive Search

(CRIS). This initial study allowed us to determine a suitable search strategy.

We initially developed a set of inclusive search terms informed by available, but limited, literature such as National Institute for Health and Care Excellence (NICE) guidelines,^14^ previous qualitative research^23,26,27^ and through discussions with clinical experts. We used an iterative approach, defining and including search terms that were sensitive enough for inclusion, but specific enough so that the search did not capture harm reduction in the context of substance misuse.

The final search criteria were: ‘(self harm* or harming self*) AND (harm reduction or harm minimisation or reduce harm or minimise harm or safe harm or less harm or stop harm or avoid harm) OR (‘alternative’ and ‘other strategies’).

#### Primary study

We then obtained data from routine electronic health records for all patients in another, larger London mental health trust. The South London and the Maudsley (SLaM) NHS Trust is a more geographically widespread secondary mental health service covering four boroughs within London: Croydon, Lambeth, Lewisham and Southwark with estimates of 1.6 million residents.^28^ The electronic health records for SLaM patients are available from 2008 using a data extraction system known as CRIS. In this process, clinical records are anonymised using a deidentification process. They provide a rich data-set for conducting clinical research with information routinely recorded such as sociodemographic and clinical characteristics, ICD-10 diagnoses and clinical record-keeping in an unstructured free-text format. For this study we obtained all patient records within SLaM from 1 January 2008 to 1 September 2019.

### Ethical approval

Ethical approval to use CRIS data in SLaM for secondary analysis was received from the Oxfordshire Ethics Committee (reference 08/H0606/71 + 5).

### Participants

#### Patient selection

**Fig. 1:**
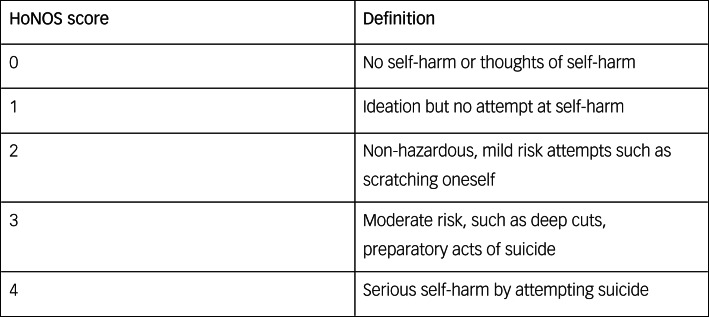
Scoring for Health of the Nation Outcome Scales ( HoNOS) Section 2 on self-harm.^29^

Our inclusion criteria were defined as any patient, of any age, who had a history of self-harm, and who had documented the use of harm minimisation as a strategy to manage self-harm in both community and in-patient settings within SLaM between the dates 1 January 2008 and 1 September 2019. To define those who self-harmed, we used section two of the Health of the Nation Outcome Scales (HoNOS);^31^ standardised routine assessment tool routinely used by clinicians throughout the UK (see Figure 1).

The HoNOS measures 12 areas of health and functioning in the context of mental health, using an ordinal coding system, and at SLaM is recorded within the electronic health records. Section two scoring is specific to self-harm but excludes accidental harm and harm as a result of injury from drug or alcohol use. To identify those who self-harmed we agreed a cut-off score of 2 or above to specify an act of self-harm.

Having defined our wider group of interest we then defined those who practised harm minimisation for self-harm by identifying mentions of self-harm within the free-text fields of the electronic health records for those patients who self-harmed and had a HoNOS score of 2 or more. Using the iterative approach outlined in the pilot study, we implemented the final search criteria as defined above.

Once patient groups were defined, we extracted clinical records on patients who self-harmed and practised harm minimisation to create a database in which each patient was linked to their free-text entry with a unique anonymised reference number. Data were extracted from unstructured progress notes, structured risk assessments and any attached documents such as correspondence or discharge summaries. A number of patients had multiple records documenting harm minimisation, in which case the first record was extracted for demographic extraction and statistical analysis; all records were included for qualitative analysis to avoid missing any mentions of method in the second or third clinical note. The initial screening process included determining whether entries were relevant in relation to our inclusion criteria, and whether the document mentioned harm minimisation specifically for self-harm rather than other clinical problems such as substance use.

#### Control group

Patients were included as a control if their notes reported self-harm at least once during the same time period but were not identified as using any harm-minimisation techniques, as per the search strategy described above.

### Data extraction

For our two comparison groups we extracted data on the following variables using CRIS: age, gender, ethnicity, marital status, employment status, socioeconomic status and comorbid diagnoses. Age in years was calculated from their date of birth in relation to either the individuals’ first HoNOS scoring (of 2 or more) or (for patients using harm minimisation) the first clinical note describing harm minimisation within the observation period. Recorded ethnicity was classified into four categories and recorded marital status was classified into three groups. Structured information on primary diagnoses using coded ICD-10 codes was collected focusing on diagnoses of personality disorder (F60*), mood disorders (F30*), anxiety disorder (F40*) substance misuse (F10–F19), eating disorder (F50*) and psychotic disorder (F20*).^30^ Any other psychiatric disorders were recorded as ‘other’ in view of small numbers in this group. We also extracted data on whether a patient had ever self-harmed with suicidal intent, requiring hospital admission; determined by a HoNOS score of 4.^31^ We also calculated the number of previous admissions to a psychiatric hospital.

### Statistical analysis

We presented descriptive statistics to describe all participants’ sociodemographic characteristics, including HoNOS scores at the time of the documented note, to determine severity of self-harm, psychiatric comorbidities, previous suicide attempts and previous hospital admissions. We used univariate logistic regression to compare the characteristics of patients who self-harmed and had reported the use of harm minimisation and those who self-harmed and do not report harm minimisation. If there was more than 20% of data missing for any variable, the variable was removed from the univariate analysis. All statistical analyses were conducted using Stata software.^32^

### Qualitative analysis

To classify the method of harm minimisation used by those in this group we used a process of content analysis, a qualitative methodology well-established in the analysis of data from electronic health records and mental health research^33^ to code free-text entries. These included accounts from patients about harm-minimisation approaches they had used, and also text describing professional advice about harm-minimisation methods as a way of managing self-harm. The key aims in coding the text were as follows.

- To classify reported methods of harm minimisation.
- To ascertain whether harm minimisation was reported as a helpful or unhelpful practice by the patient or staff member documenting the clinical note, without the coder's interpretation.

The primary researcher (C.C.), a psychiatrist, coded data to establish a classification system for each of these research questions, deriving new categories inductively using an iterative approach. A second researcher (R.S.), also a psychiatrist, independently coded 5% of included records to confirm the presence or absence of harm-minimisation practices and if present, to code and categorise the reported type of harm minimisation. We derived a Cohen's kappa value to assess interrater agreement on whether records should be included in the study, using the accepted threshold of κ = 0.749.^34^ Any disagreements were discussed between the two psychiatrists, seeking input from the wider research team for any that could not be resolved.

To address reflexivity, we used team discussions to review the emergent coding framework, reducing the potential for personal views to influence our representation of patients’ perceptions over whether harm minimisation was helpful or harmful, and of the different methods of harm minimisation reported. The analytic team included three research psychiatrists (C.C., R.S., A.P.) and a health psychologist (S.R.). The two researchers coding data each had 4–6 years of psychiatric practitioner experience in managing self-harm but no specific training in harm minimisation. The other authors had no training in harm minimisation. All authors acknowledged generally open views towards the use of harm-minimisation approaches in this context, with an awareness of potential harms and benefits.

## Results

### Pilot study

Our pilot study using Camden and Islington NHS Trust data identified 925 relevant documents included for manual coding, corresponding to 146 separate patients with documented harm minimisation and self-harm. This sample was primarily female (*n* = 109; 75%) with a mean age of 29.3 years (s.d. = 13.1). Using an iterative approach, we established a set of search terms among the team that aimed to reflect a balance of sensitivity and specificity.

### Sample size for primary study

**Fig. 2:**
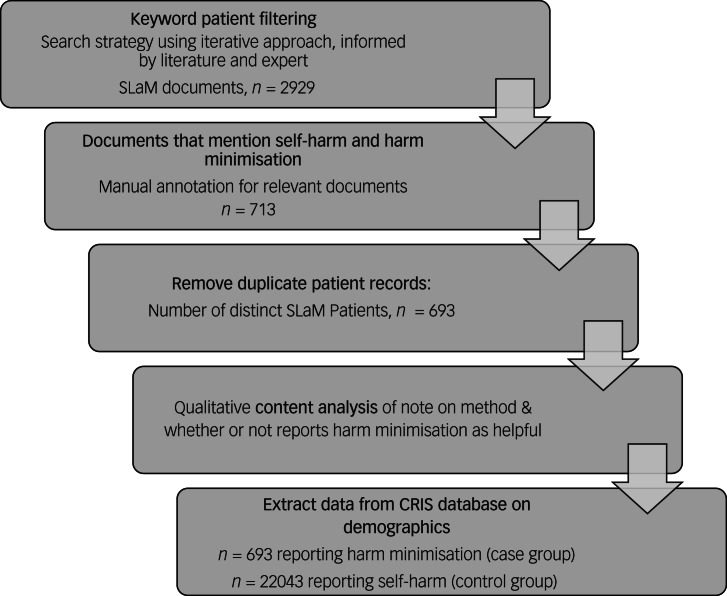
Patient identification using Clinical Records Interactive Search (CRIS) software.

Using the SLaM electronic health records for the primary study, the HoNOS scoring system identified 22 736 individuals reporting self-harm. Implementing the final search strategy identified 2929 documents of which, following initial coding, 713 documents directly reported on harm-minimisation techniques for self-harm. The remaining documents were excluded from the analysis because the cases of harm minimisation related to drug and alcohol harm minimisation rather than self-harm. After removing duplicate patients and selecting the first document reporting harm minimisation for each individual describing this, there were 693 patients (3%) (Fig. 2). Our Cohen's kappa value of 0.82 suggested a high degree of interrater reliability.^35^

### Sociodemographic and clinical characteristics

The majority of participants, 546 (79%), with records reporting harm minimisation for self-harm were female and between the ages of 16 and 25 years (Table 1).

**Table 1:** Summary of those who use and do not use harm-minimisation techniques by age group

Age group, in years	Harm minimisation *n* (%)	Control *n* (%)
Not known	84 (12.1)	1932 (8.76)
0–15	110 (15.9)	13 (<1)
16–25	288 (41.6)	5434 (24.7)
26–35	98 (14.1)	5380 (24.4)
35–44	57 (8.23)	4362 (19.8)
45–54	40 (5.77)	3287 (14.9)
≥55	16 (2.31)	1635 (7.42)
Total	693	22 043

Information on gender, marital status, ethnicity, employment, scale 2 of the HoNOS scoring system for self-harm, previous self-harm with suicidal intent, and prior admissions, are provided in Table 2, together with unadjusted odds ratios for comparisons of patients with documented harm minimisation and those not using harm minimisation (the control group).

**Table 2:** Sociodemographic characteristics of patients by use of harm minimisation

Variables	Patients using harm minimisation (harm-minimisation group) (*n* = 693)	Patients not using harm minimisation (control group) (*n* = 22 043)	Unadjusted odds ratio (95% CI)	*P-*value
Total, *n* (%)	693 (3.1)	22 043 (96.8)	--	--
Age, years: mean (s.d.)	24.4 (12.0)	36.0 (12.8)	0.89 (0.88–0.90)	<0.001
Gender, female: *n* (%)	546 (78.8)	12 474 (56.6)	2.92 (1.60–3.39)	<0.001
Marital status, *n* (%)				
Single	468 (67.5)	13 588 (61.6)	ref	ref
Married	43 (6.20)	3083 (14.0)	0.40 (0.30–0.55)	<0.001
Divorced/separated/widowed	44 (6.35)	2204 (10)	0.58 (0.42–0.79)	0.001
Not known	138 (19.9)	3168 (14.4)	Not included	Not included
Ethnicity, *n* (%)				
White	506 (73.0)	13 166 (59.7)	ref	ref
Asian	34 (4.91)	1129 (5.12)	0.78 (0.55–1.11)	0.18
Black	67 (9.67)	4151 (18.8)	0.42 (0.32–0.54)	<0.001
Others/mixed	40 (5.78)	2046 (9.28)	0.51 (037–0.70)	<0.001
Not known	46 (6.64)	1551 (7.0)	Not included	Not included
Employment, *n* (%)				
Not known	591 (85.3)	16 986 (77.1)	n/a^a^	--
Employed	44 (6.35)	1428 (6.48)	--	--
Unemployed	46 (6.64)	3334 (15.1)	--	--
Other (e.g. retired/homemaker/students)	12 (1.73)	295 (1.34)	--	--
HoNOS score reporting at time of clinical note (mean), *n* (%)				
Not recorded	383 (55.3)	--	--	--
1	59 (8.51)	0	n/a	--
2	110 (15.9)	12 459 (56.5)	0.64 (0.45–0.91)	0.02
3	100 (14.4)	6612 (30.0)	1.10 (0.76–1.58)	0.62
4	41 (5.92)	2972 (13.5)	ref	--
Suicide attempt, *n* (%)	100 (14.4)	6612 (30.0)	0.39 (0.32–0.49)	<0.001
Number of previous admissions				
Mean (s.d.)	2.30 (2.85)	1.89 (1.76)	1.2 (1.13–1.21)	<0.001
Range	1–17	1–22	--	--

n/a, not applicable; HoNOS, Health of the Nation Outcome Scales; ref, reference.

a. As a result of the high number of missing values.

Among patients who self-harmed, those with documented harm minimisation were significantly younger than those without documented harm minimisation (mean age 24.4 years *v*. 36.0 years, odds ratio (OR) = 0.89, 95% CI 0.88–0.90, *P* < 0.001), and were also more likely to be female (79% *v*. 57%, OR = 2.92, 95% CI 1.60–3.39, *P* < 0.001) (Table 2).

There were no significant group differences in ethnicity, employment status or socioeconomic group. Among those using harm minimisation there were fewer reports of serious self-harm resulting in hospital admission, compared with those not using harm minimisation (14.4% *v*. 30.0%, OR = 0.39, 95% CI 0.32–0.49, *P* < 0.001), but a significantly higher number of previous psychiatric admissions (mean 2.30 *v*.  1.89, OR = 1.2, 95% CI 1.13–1.31, *P* < 0.001).

Patients with documented harm minimisation were significantly more likely to have a diagnosed affective or anxiety disorder (*n* = 186, 26.6% *v*. *n* = 3676, 16.7%, OR = 2.46, 95% CI 1.98–3.06, *P* < 0.001), eating disorder *n* = 32, 4.62% (control *n* = 433, 1.96%, OR = 3.63; 95% CI  2.55–5.39; *P* < 0.001), but were significantly less likely to have a psychotic disorder, F20, *n* = 25, 3.61% (control *n* = 2336, 10.6%, OR = 0.53, 95% CI 0.34–0.81; *P* = 0.03) (Table 3). There were no significant group differences in personality disorder diagnoses *n* = 27, 3.9%, (control *n* = 950, *n* = 4.31%, OR = 1.4, 95% CI 0.92–2.11, *P* = 0.11).

**Table 3:** Comorbid diagnosis

Variables	Patients using harm minimisation, *n* (%) (*n* = 693)	Patients not using harm minimisation, *n* (%) (*n* = 22 043)	Unadjusted odds ratio (95% CI)	*P-*value
Affective and anxiety disorder	184 (26.6)	3676 (16.7)	2.46 (1.98–3.06)	<0.001
Personality disorder	27 (3.90)	950 (4.31)	1.40 (0.92–2.11)	0.11
Eating disorder	32 (4.62)	433 (1.96)	3.63 (2.55–5.39)	<0.001
Psychotic disorder	25 (3.61)	2336 (10.6)	0.53 (0.34–0.81)	0.03
Other psychiatric diagnoses, including substance misuse and developmental	271 (39.1)	7067 (32.1)	1.89 (1.54–2.31)	<0.001
Not known	154 (22.2)	7581 (34.4)	n/a	--

### Content analysis: descriptions of harm minimisation

Our content analysis identified a range of harm-minimisation methods for self-harm described within patients’ routine clinical notes, grouped into four categories. These are set out below with illustrative quotes, changing names to unrelated letters. Some patients reported more than one harm-minimisation technique, the number of individuals reporting more than one method is *n* = 86. We counted these as separate approaches in our classification, and the four categories below (substitution, simulation, defer and avoid, and damage limitation) present numbers and percentages in relation to the total of all mentions of harm minimisation, with a denominator of *n* = 822 (patient, *n* = 693). The most frequent form of harm minimisation was ‘substitution’, representing 51.9% (427/822) of the 822 mentions of harm minimisation. In cases where harm minimisation was implemented, records were frequently missing any details about the method of harm-minimisation technique being used. A total of 193 of the 822 mentions (23.5%) could not be classified based on the limited information reported.

#### Substitution

The first category of ‘substitution’ described strategies for harm minimisation in the context of self-harm that replace or replicate pain, for example using an elastic band, cold water or ice. It also included approaches to substituting the pain with alternative methods of frustration release, such as punching a pillow. Individuals reported that they used substitution methods as a coping mechanism to re-direct their urge to self-harm. In total there were 427 (51.9%) mentions of substitution methods.

‘G has identified that using ice or rubber bands if she felt the urge to self-harm was extremely strong, she could do so.’

‘We discussed alternative harm reduction forms of “self-harm” e.g. holding an ice cube in your hand or using an elastic band at the wrist instead of cutting herself.’

#### Simulation

The ‘simulation’ category describes simulating the process of self-harm such as using a red pen or crushing blackcurrants onto one's skin, to simulate blood. In total there were 70 (8.51%) mentions of simulation techniques:

‘She still has the urge [to self-harm] but is using alternatives such as drawing on her arm with red pen.’

‘strategies that she could use instead of self-harm such as red marker pen.’

#### Defer and avoid

A third category: ‘defer or avoid’ describes methods that could delay self-harm such as removing access to any sharp objects. It also includes methods that aim to avoid the risk of physical damage, such as providing education about anatomical positioning on where it is safest to self-cut. This was described as being used in both an in-patient context, as well as at home with support from friends. There were 58 (7.10%) mentions of defer and avoid methods:

‘H was always careful to avoid a big blood vessel.’

‘Discussed harm reduction plan – give scalpel to house mate and remove sharps.’

### 

#### Damage limitation

Our fourth category was termed ‘damage limitation’. This includes mentions of wound care to minimise the risk of infection once the self-harm has been inflicted. This category includes some of the more controversial methods such as recommending ‘clean’ blades, however, we found very few records reporting this. There were 74 (9.0%) mentions of damage limitation usage:

‘I have provided E with literature/information about cleaning wounds following self-harm.’

‘F had sterile bandages/razors etc to minimise harm should she feel overwhelmed at self-harm.’

### 

#### Unclassified

Finally, we created a fifth category for those notes mentioning no details of the type of harm minimisation, apart from that harm minimisation was being used to help manage self-harm. We included 193 (23.5%) notes of harm minimisation in this category:

‘I agreed to a safety contract about not making any attempts to harm herself and we discussed some harm minimisation strategies as part of the appointment.’

‘J requested for further information regarding harm minimisation for self-harm.’

### Content analysis: perceived helpfulness of harm minimisation

Using a content analysis approach, we identified that 638 (92.1%) of the 693 individuals reporting the use of harm minimisation, described this in a manner that we coded as helpful, whereas 55 (7.94%) described this in a manner that we coded as unhelpful.

Responses were coded as ‘helpful’ if patients perceived the use of harm minimisation as an effective method of managing their urge to self-harm, as well as those where patients viewed professionals’ use of education in harm minimisation as a therapeutic technique to manage their urge to self-harm. Examples were as follows:

‘X copes with urge to superficially self-harm using a rubber band against his skin.’

‘Q specifically stated that pinching herself is a useful alternative to acting on her more severe ideas of harming herself.’

Responses coded as reporting the technique as ‘unhelpful’ suggested that harm-minimisation strategies were either ineffective or had even escalated self-harm. Examples were as follows:

‘S said alternatives offered to her in the past have been ineffective e.g. plunging her head in icy water, pushing walls etc.’

‘L commented that stuff like the rubber bands escalates into self-harm.’

‘E was advised to use red ice cubes to help her urges which she tried but stated they had no effect.’

## Discussion

### Main findings

A small minority (less than 3%) of patients who self-harm, under the care of a London mental health trust, were documented as having used harm minimisation, although the majority (92%) of this group described it as helpful. It is important to highlight that those finding harm minimisation ‘unhelpful’ reported the techniques as either ‘ineffective’ or a small minority reported a risk of harm minimisation escalating into self-harm, highlighting potentially harmful effects of harm minimisation.

Of those that did report harm minimisation, more than half reported using what we classified as substitution techniques such as elastic bands, ice and cold water to reduce harm. We found that a quarter of the records lacked sufficient detail of the specific approach to harm minimisation used, which could suggest inadequate recording practices or some lack of familiarity among staff over documentation of the approaches available.

Among all patients who had a history of self-harm, those who reported the use of harm-minimisation strategies were more likely to be younger in age, single, of White ethnicity and female. They were most likely to be aged between 16 and 25 years; the age at which patients may be most likely to be active online with exposure to educational materials shared online about harm minimisation.^36^ Patients with records reporting the use of harm-minimisation techniques were also more likely to have had previous psychiatric admissions. These treatment episodes will have increased their contact with other patients or professionals, providing opportunities to hear about harm minimisation, theoretically increasing the likelihood of its use. However, they were less likely to have previously self-harmed with suicidal intent. This warrants further investigation of the role of harm minimisation in those with intermittent suicidality.

### Findings in the context of other literature

Similar to our findings, previous research reports low prevalence of harm-minimisation techniques being used by people who self-harm. In a recent British study only 0.9% of participants who self-harm in a community and clinical sample reported the use of harm minimisation and the majority of these (86%) reported use of a rubber band.^26^ These findings seem to suggest that in clinical samples of people who self-harm, a small minority of individuals use harm minimisation, or are prepared to disclose that they do. Where people who self-harm do report using harm minimisation, the most common approach reported aligns to that of our category of ‘substitution’ techniques, such as a rubber band or ice to substitute the pain. This is consistent with the views of professionals and carers over which techniques are regarded as ‘safer’ or less controversial.^27^

Our classification of harm-minimisation techniques has some differences from the taxonomy developed in a previous British study analysing individuals sampled from community and clinical settings.^26^ Our category ‘substitution’ includes replacing the pain with alternative methods of frustration release, such as punching a pillow, alongside Wadman's ‘sensation’ techniques such as ice or a rubber band.

Our ‘defer and avoid’ category includes methods that delay self-harm such as removing access to sharp objects and education about anatomical positions that would avoid severe physical damage inflicted by the self-harm. Our fourth category ‘damage limitation’, includes mentions of wound care to minimise the risk of infection; this group echoes Wadman's own ‘damage limitation’ category, which also ensures that aftercare of self-harm minimises any potential complications. The categorisation of the different harm-minimisation approaches is subject to debate with a consideration of wider clinical context, as some methods involving sensory stimulation may also be viewed as grounding techniques that are used to bring people out of dissociative states.^26^ It would be beneficial to develop a framework of harm-minimisation approaches for self-harm that can be applied consistently across clinical and research settings.

Our findings suggest that the majority of people with records reporting harm minimisation find it helpful; this is consistent with previous studies reporting patients’ and practitioners’ positive opinions on harm minimisation and the need to be flexible when approaching self-harm behaviour.^24,27^ However, the British study described above reported that 7% of participants using harm-minimisation approaches perceived them as ineffective or unhelpful.^26^ Another, recent UK-based study interviewed 126 adolescents in the community aged between 11- and 21-years-old and found a third perceived harm minimisation as helpful, a third were indifferent and a third perceived harm minimisation as unhelpful.^37^ These differing findings suggest that setting, quality of clinical training in harm minimisation and clinical severity of self-harm may be key influences on treatment preferences, although these differences may be an artefact of the question used in research studies.

Previous studies have reported patients’ concerns over harm minimisation contributing to an increase in tolerance to pain and sensation seeking.^18,27^ Other work describes the concerns of practitioners and carers regarding the potential for harm minimisation to escalate the severity of self-harm over time.^20,38^ Specific concerns were raised over the potential for anatomical knowledge to increase the risk of a more severe and potentially fatal injury.^20,22^ However, other practitioners felt that harm minimisation was able to empower patients and increase autonomy, albeit with caveats around the need for careful monitoring.^20,39^

As with professionals, adolescents appear to be divided in their opinions over whether harm minimisation is helpful or not, and this appears to be context-dependent harm minimisation.^26,37^ Research also describes conflicting opinions on this among carers, including particular concerns about the risks of providing vulnerable young people with anatomical information.^27^ In this study the majority of those using harm minimisation found it to be helpful, but this was in the context of a very low prevalence of patients who self-harm reporting the use of harm minimisation *per se*.

### Strengths and limitations

To our knowledge, this study is the first to analyse routine secondary mental healthcare data specifically to investigate the use of harm minimisation in the clinical management of self-harm. Our study provides a valuable insight into the sociodemographic and clinical characteristics of patients in a large mental health trust, diverse in geography, social deprivation and ethnicity. Our sample represents the largest to date among studies researching harm minimisation for self-harm and use of the CRIS tool provided us with comprehensive quantitative and qualitative data for each patient. Our access to free-text fields within electronic health records provided us with important insights into the harm-minimisation approaches used in clinical practice. Our collaborative team approach to independent data coding and interpretation enhanced the validity of our categorisation of harm-minimisation practices. Our use of discussions to address reflexivity reduced the potential for our own views to influence the coding framework.

Our results should be considered in light of several limitations. It was challenging to remain as inclusive as possible when applying iterative search strategies and manually coding the notes, within the limits of realistic volumes of data to analyse. Using the search terms ‘harm reduction and harm minimisation’ produced a large number of irrelevant records, the majority relating to patients using addiction services, who also reported self-harm, as often the two presentations co-occur. Therefore, it was difficult to exclude such cases from the search without risking a search strategy that was not sufficiently inclusive.

Our use of routine clinical notes, rather than data collected primarily to address this research question, meant that we encountered a high proportion of missing data for specific sociodemographic variables. We also acknowledge the potential for professionals’ anxiety about harm minimisation, in view of some of the more controversial methods, to result in underreporting of harm minimisation in electronic health records. There may be several reasons for these low rates. First, the use of harm minimisation may not be routinely probed by clinicians, and in not asking questions about harm-minimisation clinical records may under-record its true prevalence. Second, clinicians may be reluctant to use harm minimisation for self-harm because of concerns about safety, a lack of local or national guidelines, or the lack of evidence for its efficacy. Third, a lack of time or documentation may restrict the information available in clinical records that describe harm-minimisation approaches to self-harm. The quarter of records in which the approach used was not apparent suggests a need for clearer recording of harm-minimisation strategies within routine clinical notes. Finally, although SLaM's mental health Trust covers four diverse London boroughs, findings from a London setting may not be generalisable to the rest of the UK or to population samples.

### Clinical and research implications

Although very few patients in our clinical sample were recorded as using harm minimisation, and this was a relatively distinct demographic, a very high proportion found it helpful. This suggests, in the context on minimal clinical guidelines, that there might be scope to introduce this practice more widely. However, previous studies have described uncertainties and anxiety among health professionals about the use of harm minimisation. Clearly defined policies based on evidence of acceptability and effectiveness would provide practitioners with the confidence to try harm-minimisation approaches with patients, particularly where local consensus was achieved over training needs and organisational approach.

To achieve this, an evaluation of harm-minimisation approaches for self-harm is needed, in order to determine what approaches are helpful (or not), and for whom. We need qualitative studies in both community and in-patient settings to explore the acceptability of different harm-minimisation techniques among a range of patients, carers and practitioners, including perceived harms. Randomised controlled clinical trials are also needed to determine if harm minimisation achieves a reduction in physical damage, distress, or the frequency of self-harm or suicidality, and which methods would be most effective at achieving this, as well as which patient characteristics predict treatment response and potential harms. Without this evidence base and guidance on harm-minimisation implementation, it is likely that uncertainties about the potential clinical value of harm-minimisation approaches will persist.

## Data availability

Data is available on request due to privacy/ethical considerations. The data accessed by CRIS remain within an NHS firewall and governance is provided by a patient-led oversight committee. Subject to these conditions, data access is encouraged and those interested should contact the CRIS academic lead via the corresponding author.
